# MicroRNAs Instruct and Maintain Cell Type Diversity in the Nervous System

**DOI:** 10.3389/fnmol.2021.646072

**Published:** 2021-04-29

**Authors:** Norjin Zolboot, Jessica X. Du, Federico Zampa, Giordano Lippi

**Affiliations:** ^1^The Scripps Research Institute, La Jolla, CA, United States; ^2^Department of Neurosciences, University of California, San Diego, San Diego, CA, United States

**Keywords:** microRNA, cell type, CNS – central nervous system, neural progenitor, cell fate, neuron, glia, cell diversity

## Abstract

Characterizing the diverse cell types that make up the nervous system is essential for understanding how the nervous system is structured and ultimately how it functions. The astonishing range of cellular diversity found in the nervous system emerges from a small pool of neural progenitor cells. These progenitors and their neuronal progeny proceed through sequential gene expression programs to produce different cell lineages and acquire distinct cell fates. These gene expression programs must be tightly regulated in order for the cells to achieve and maintain the proper differentiated state, remain functional throughout life, and avoid cell death. Disruption of developmental programs is associated with a wide range of abnormalities in brain structure and function, further indicating that elucidating their contribution to cellular diversity will be key to understanding brain health. A growing body of evidence suggests that tight regulation of developmental genes requires post-transcriptional regulation of the transcriptome by microRNAs (miRNAs). miRNAs are small non-coding RNAs that function by binding to mRNA targets containing complementary sequences and repressing their translation into protein, thereby providing a layer of precise spatial and temporal control over gene expression. Moreover, the expression profiles and targets of miRNAs show great specificity for distinct cell types, brain regions and developmental stages, suggesting that they are an important parameter of cell type identity. Here, we provide an overview of miRNAs that are critically involved in establishing neural cell identities, focusing on how miRNA-mediated regulation of gene expression modulates neural progenitor expansion, cell fate determination, cell migration, neuronal and glial subtype specification, and finally cell maintenance and survival.

## Introduction

Understanding the biological basis of the vast cellular diversity found in the nervous system remains a high priority for neuroscience research. Recent advances in single-cell transcriptomics have enabled the exploration of neural cell diversity with increasing spatial and temporal resolution, generating unprecedented quantitative and comprehensive datasets characterizing the transcriptomes, morphology and electrophysiology of neuronal subtypes (Zeisel et al., 2015, 2018; Shekhar et al., 2016; Tasic et al., 2016, 2018; Saunders et al., 2018; Gouwens et al., 2019). Moreover, combining single-cell RNA sequencing with techniques such as patch-clamp has allowed scientists to establish direct correspondence between transcriptomic, morphological and physiological datasets leading to a more integrative and multimodal approach of classifying cell types (Cadwell et al., 2016; Fuzik et al., 2016; Földy et al., 2016; Scala et al., 2019; Gouwens et al., 2020). However, the molecular mechanisms that instruct the emergence of cell diversity remain elusive.

There is increasing evidence that microRNAs (miRNAs) can act as key regulators of cellular identity. miRNAs are non-coding RNAs that function as post-transcriptional repressors of mRNA expression. A distinctive feature of miRNAs is that evolution has favored a continual expansion in the miRNA repertoire with increasing number of distinct cell types in an organism (i.e., biological complexity) across metazoans. This is in clear contrast to protein-coding genes, whose number show no correlation to biological complexity. Once miRNAs are added to metazoan genomes and integrated into gene regulatory networks, they are strongly conserved in primary sequence and rarely secondarily lost (Heimberg et al., 2008; Kosik, 2009; Liu et al., 2013). These features strongly suggest that cellular diversity might arise from increasingly sophisticated regulation of gene expression by non-coding RNAs, and in particular by miRNAs.

The expression patterns of miRNAs in the brain show an impressive specificity for distinct developmental stages, brain regions and cell types. A single miRNA is capable of regulating hundreds of different targets and these targets can also vary according to specific cell types and developmental stages (He et al., 2012; Jovičić et al., 2013; Nowakowski et al., 2018). This suggests that the brain utilizes differential miRNA expression and target regulation to establish and maintain cellular diversity. Indeed, during the development of a cell type, miRNAs are known to sharpen developmental stage transitions by repressing residual transcripts specific to the previous stage. Once the cells have achieved a mature differentiation state, miRNAs confer robustness to the developmental decision by reducing fluctuations in gene expression and restricting protein levels within a range of values that maintain cell identity (Ebert and Sharp, 2012).

The dynamic expression patterns of miRNAs, their ability to facilitate developmental transitions and fine-tune protein levels, their conservation as well as their evolutionary expansion with increasing biological complexity all make miRNAs well-suited to instruct and maintain the astonishing cellular diversity found in the nervous system. In this review, we discuss current evidence supporting critical roles for miRNAs in determining cell identity across their developmental trajectory. We describe miRNAs and their targets that are critical throughout neural development from neurulation to neural progenitor expansion, fate determination, neuronal and glial subtype specification, and finally maintenance and survival of these cell types. Furthermore, we also discuss how miRNAs regulate migration, lamination, morphology, and functional connectivity of neurons and glia, all aspects that are integral to cellular identity. A summary of the miRNAs and targets involved in all of these processes are listed in Table 1.

**TABLE 1:** Summary of the most important miRNAs that instruct the identity of multiple cell types in the nervous system.

CELL type	Developmental processes	miRNA	Known targets	References
Neural progenitors	Proliferation	let-7, miR-125, miR-9, miR-137	lin-28	Rybak et al., 2008
		let-7, miR-9, miR-137	Nr2e1	Zhao et al., 2009
	Proliferation, cell cycle	miR-2115	Orc4	Nowakowski et al., 2018
		miR-302a-d		Parchem et al., 2015
	Proliferation, differentiation	miR-20a, miR-20b, miR-23a	CyclinD1	Ghosh et al., 2014
		miR-934		Prodromidou et al., 2020
		miR-486a/b-5p		Dori et al., 2020
	Neuronal differentiation	miR-124, miR-9	REST, BAF complexes	Yoo et al., 2011; Lee et al., 2018
		miR-124	Ptbp1	Makeyev et al., 2007

Intermediate progenitors	Differentiation	miR-92b	Tbr2	Nowakowski et al., 2013

Retinal progenitors	Neurogenesis, differentiation	let-7, miR-125, miR-9	Prtg, Lin28b	La Torre et al., 2013

Olfactory progenitor cells	Neurogenesis, survival	miR-200a-c, miR-429, miR-141	Mash1	Choi et al., 2008

Adult neurogenic progenitors	Proliferation, progenitor identity	miR-184, miR-34a	Numbl	Shen et al., 2002; Fineberg et al., 2012
	Neuronal differentiation	miR-124	Sox9	Cheng et al., 2009

Cortical pyramidal neurons	Laminar identity	miR-128, let-7b, miR-9		Shu et al., 2019
	Migration	mi-129-3p/5p	Fmr1	Wu et al., 2019
		miR-396-3p, miR-496, miR-543	Cdh2	Rago et al., 2014
	Dendritic outgrowth	miR-101	Slc12a2, Ank2, Kif1a	Lippi et al., 2016
		miR-9		Giusti et al., 2014

Adult-born hippocampal neurons	Migration	miR-19	Rapgef2	Han et al., 2016
	Dendritic outgrowth	miR-132, miR-212		Vo et al., 2005; Magill et al., 2010
		miR-19		Han et al., 2016

Cajal–Retzius cell	Differentiation	miR-9	Foxg1	Shibata et al., 2011

Corticospinal motor neurons	Differentiation, axon growth	miR-409-3p	LMO4	Diaz et al., 2020

Dopamine neurons	Differentiation, survival	miR-133b	Pitx3	Kim et al., 2007
		miR-200c	Zeb2	Yang et al., 2018

Spinal motor neurons	Differentiation, survival	miR-218	Kcnh1	Thiebes et al., 2015; Reichenstein et al., 2019

Dorsal root ganglion neurons	Axon growth	miR-132	Ras1	Hancock et al., 2014

Retinal ganglion cells	Axon growth	miR-182	Cfl1	Bellon et al., 2017

Retinal photoreceptors	Differentiation, morphology	miR-183/96/182 cluster		Busskamp et al., 2014

Olfactory interneurons	Migration, dendritic outgrowth	miR-125		Åkerblom et al., 2014

Olfactory dopamine neurons	Differentiation	miR-7a	Pax6	de Chevigny et al., 2012

Mechanosensory neurons	Subtype specification	miR-183/96/182 cluster	Shox2, Cacna2d1/2	Peng et al., 2018

Microglia	Activation state	miR-128, miR-124	Cebpa	Yang et al., 2017

Oligodendrocytes	Differentiation	miR-219, miR-338	Sox6, Hes5, Zfp238	Dugas et al., 2010; Zhao et al., 2010
		miR-7a	Pax6, NeuroD4	Zhao et al., 2012
		miR-23	LmnB1	Lin and Fu, 2009
	Survival	miR-17/92 cluster	Pten	Budde et al., 2010

Schwann cells	Differentiation, proliferation	miR-34a	Notch1, Ccnd1	Viader et al., 2011
		miR-140	Egr2	Viader et al., 2011

Astrocytes	Differentiation, proliferation	miR-31	lin-28	Meares et al., 2018
		miR-125b		Pogue et al., 2010

*For each cell type, the developmental processes and the relevant miRNAs are listed. In addition, known targets of those miRNAs and the main references are indicated (e.g., in human neural progenitors, the timing and duration of the cell cycle is regulated by the great ape-specific miR-2115 through the repression of the DNA replication regulator ORC4).*

## Neurogenesis and Neuronal Fate

Developmental programs generate distinct cell types as opposed to continuous diversity by making distinct lineage decisions, which sequentially narrow the range of possible forms and functions. Early lineage decisions, including those that affect progenitor cells, have enormous power over the developmental trajectories a newly born cell can follow. Progenitor state, which is known to change over developmental time and in response to signaling, sets daughter cells along the path to a neuronal fate or glial fate, biases them to specify into early-born or late-born cell types, and determines their responsiveness to extrinsic maturation cues (Telley and Jabaudon, 2018). In this section, we review how miRNAs affect early stages of cell identity by controlling multiple aspects of progenitor development, from expansion and maintenance of the stem cell population to the production of a range of neuronal subtypes in several different progenitor niches.

### Proliferative Progenitors

It is common to interrogate the overall function of miRNAs by examining phenotypes induced by removal of all miRNAs. Mature, functional miRNAs are processed from precursor miRNAs by the endonuclease activity of the enzyme DICER. Thus, DICER knockout prevents miRNA biogenesis and eventually results in functional ablation of the vast majority of miRNAs (Davis and Hata, 2009). While global miRNA ablation is embryonic lethal (Bernstein et al., 2003), conditional and inducible DICER knockouts have provided much insight on the roles miRNAs play in different cell types at each stage of development. Recent reports suggest that well-known miRNA biogenesis enzymes participate in regulatory mechanisms independent of miRNA function, for example by cleaving other classes of RNAs or even regulating DNA repair (Pong and Gullerova, 2018). Thus, DICER knockout studies reflect a ceiling effect, including the effects of removing almost all miRNAs as well as miRNA-independent functions of DICER. Probing the mechanisms of developmental gene regulation at finer resolution will necessarily require disentangling phenotypes and consideration of non-canonical functions of miRNA biogenesis enzymes, but these experiments serve as a starting point for investigating specific miRNA functions, as illustrated by the next few examples.

**FIGURE 1:**
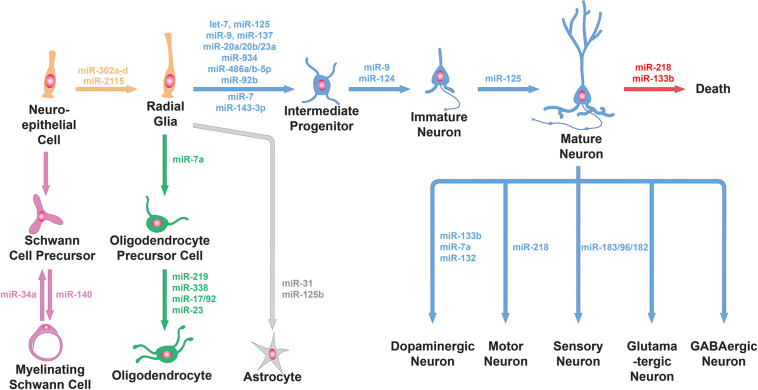
miRNA functions in neural cell lineage. miRNAs are involved in the generation of progenitors from neuroepithelial cells, the differentiation of progenitor cells into neuronal or glial cells, the further specialization of neuronal cells and glial cells into specific subtypes and neuronal survival. Specific examples of miRNAs involved in these processes (e.g., miR-9 and miR-124 for neuronal differentiation, miR-31 for astrocyte differentiation, miR-218 for the specification and survival of motor neurons and miR-34a for Schwann cell dedifferentiation) are shown here.

A study examining miRNA ablation via DICER knockout in Emx1-expressing cortical cells and progenitors found no defect in proliferation or neurogenesis, only defects in later differentiation and survival (de Pietri Tonelli et al., 2008). However, miRNAs may persist long after DICER knockout begins, meaning that this manipulation may not effectively probe miRNA function in early progenitors. miRNA ablation at an earlier timepoint—using Rx-Cre-driven conditional knockout of DICER, which starts at E7.5—caused overt structural defects in the neuroepithelium by disrupting adherens junctions and inducing hyperproliferation of radial glial cells (Fernández et al., 2020). Part of the above phenotype seem to be mediated by miR-302a-d, a member of a miRNA family highly implicated in regulating cell cycle in embryonic stem cells. miR-302a-d is essential to successful brain development in mammals. Knockout of this single miRNA caused profound dysregulation of early neural progenitors: increased proliferation, decreased apoptosis, and premature differentiation of progeny neurons (Figure 1, top-left). Ultimately, over-expansion of neural progenitors thickened the neuroepithelium, preventing closure of the neural tube and leading to embryonic lethality (Parchem et al., 2015). Recently, an intriguing hypothesis has been put forward, suggesting that primate-specific miRNAs selectively affect this process. Timing and duration of the cell cycle in radial glia progenitors is regulated by the great ape-specific miR-2115 through repression of the DNA replication regulator ORC4 (Figure 1, top-left). This interaction could provide insight on the evolution of progenitor expansion mechanisms in human brain development (Nowakowski et al., 2018).

While studying the roles of these miRNAs, it is important to remember that miRNAs function in complex networks with their targets, other miRNAs, and other non-coding RNAs. For example, one or multiple miRNAs may target multiple aspects of a cellular pathway or biological process in order to cooperatively regulate neuronal progenitors. This allows miRNAs to substantially boost the magnitude of repression and fine tune the timing of regulation in response to several intersecting cues. In addition, miRNAs and their targets may form regulatory loops for controlling the timing of critical transitions and strongly reinforcing fate decisions. The lin-28 gene supports pluripotency in proliferating stem cells, where it is highly expressed, and simultaneously suppresses the production of mature let-7 and miR-125 (Rybak et al., 2008). When lin-28 expression begins to fall as the cells differentiate, let-7 and miR-125 escape repression and further repress lin-28 translation, forming an auto-regulatory loop that quickly changes gene expression to reinforce the fate decision. Let-7 has also been shown in cancer cell lines to repress multiple other factors involved in proliferation (Johnson et al., 2007; Dong et al., 2010), and in neurons cooperates with miR-9 and miR-137 to repress TLX, again inhibiting progenitor proliferation by downregulating Wnt signaling (Zhao et al., 2009; Sun et al., 2011). Biogenesis of miR-9 and miR-137 is repressed by TLX in unique positive feedback loops. Thus, miRNAs such as let-7 illustrate the complex molecular circuitry required to regulate progenitor proliferation and fate decisions with temporal precision.

MicroRNA regulation of the progenitor pool is the first step required for proper development of the brain architecture and thus foundational to maintaining proper developmental trajectories. Below, we discuss the following stages that are necessary to setting up the cell diversity in the brain.

### Cortical and Subcortical Neurogenesis

Neuronal fate appears as progenitors switch from expanding the progenitor pool to producing terminally differentiated neurons or neurogenic progenitors with restricted potency. miRNAs are crucial regulators of these processes. For example, cyclin D1, a key component of cell cycle regulation that affects the balance between progenitor proliferation and neuronal differentiation, both regulates and is regulated by miR-20a, miR-20b, and miR-23a, forming a regulatory loop controlling the transition to neurogenesis and differentiation (Ghosh et al., 2014). Other miRNAs are necessary for progenitor proliferation. miR-486a/b-5p is downregulated as progenitors transition from self-replenishing proliferation to neurogenesis; ectopic expression in radial glia prolongs the proliferative stage, leading to over-expansion of the progenitor pool at the expense of neuron number (Dori et al., 2020). Experiments in human induced pluripotent stem cells (iPSCs) have identified the primate-specific miR-934 as another potential regulator of this transition, with miR-934 activity during neural induction of stem cells correlating to neurogenesis and decreased numbers of proliferative progenitors (Prodromidou et al., 2020).

Adult neurogenesis requires miRNAs to do the opposite: stably maintain progenitor fate to allow proliferation throughout life. Adult stem cells undergo asymmetric divisions to repopulate the stem cell pool while producing more differentiated progenitors, fated to produce differentiated neurons. miR-184 and miR-34a upregulate Notch signaling in the daughter cell fated for neuronal differentiation by targeting the Notch repressor Numbl (Shen et al., 2002; Fineberg et al., 2012). Meanwhile, this pathway is not active in the other daughter cell, which maintains its stem cell identity and tightly controls further proliferation. Thus, miRNAs play critical roles in controlling progenitor fate, whether that means transitioning to terminal differentiation during development or maintaining stem cell identity in adulthood.

The function of a miRNA can vary depending on the progenitor population, co-expressed factors, and developmental timepoint. miR-9 provides an illustrating example of this complexity. miR-9 overexpression at E11.5, at the start of neurogenesis, increased differentiation of progenitors and caused overproduction of Cajal-Retzius cells, one of the earliest cortical cell types produced and a key director of cortical lamination (Shibata et al., 2008). This stems from dysregulation of Foxg1, a transcription factor thought to promote proliferation and suppress early differentiation, in the cortical hem. However, by E16.5 Foxg1 seems to escape the repressive influence of miR-9; *in vitro* experiments suggest that this is mediated by co-expression of Foxg1 and Elavl2, an RNA binding protein that may block miR-9 from targeting Foxg1’s 3′UTR. At these ages, miR-9 seems to decrease cortical progenitor proliferation by repressing other transcription factors such as Pax6, Meis2, and Nr2e1 (Shibata et al., 2011). At the midbrain-hindbrain boundary (MHB), miR-9 expression is repressed in progenitors of since these cells must resist differentiation in order to signal in cell fate decisions in surrounding areas. Ectopic miR-9 expression caused these progenitors to become neurogenic and lose MHB markers (Leucht et al., 2008). miRNA function is modified by changes in the cell type-specific and developmentally regulated network of co-expressed factors, allowing more nuanced control of progenitor and neuronal fate.

During these progenitor transitions, miRNAs may also be regulated by other non-coding RNAs, which can sequester miRNAs at repeated miRNA binding sites—so-called ‘miRNA sponges’—thus blocking their function on target mRNAs. miR-7 and the stem cell-expressed lncRNA Cyrano form an autoregulatory loop, with miR-7 overexpression in zebrafish zygotes causing defects in early brain development that are rescued by expression of a miR-7-insensitive mutant Cyrano (Smith et al., 2017; Sarangdhar et al., 2018). Meanwhile Cyrano represses miR-7 by triggering target-directed miRNA degradation, which occurs when miRNAs bind sites with high complementarity extending beyond the seed region into the 3′ region (Han et al., 2020). The primate long non-coding RNA (lncRNA) LncND binds miR-143-3p to prevent repression of Notch signaling and promote proliferation (Rani et al., 2016). Ectopic expression of LncND in mouse embryos during early cortical development significantly increased the population of radial glia and decreased numbers of more differentiated intermediate progenitors (IPs). The human circRNA CDR1as sequesters miR-7; both miR-7 knockdown and exogenous CDR1as expression lead zebrafish to exhibit underdeveloped midbrains (Memczak et al., 2013). The complex loops and network relationships miRNAs form with other classes of ncRNAs contributes to finely tuned control of neurogenesis and potential species-specific mechanisms of brain development.

Neurogenic progenitors can also transition through progenitor subtypes and gradually undergo multipotency restriction. Cortical progenitors—radial glia—divide to generate IPs, which increase the neurogenic capacity of the progenitor pool. miRNAs seem to negatively regulate IP production, in particular through repression of the IP marker Tbr2 by miR-92b (Nowakowski et al., 2013; Figure 1, top-left). And when miRNAs are ablated in Emx1-expressing differentiated progenitors, these cells fail to switch from producing deep layer neurons to upper layer neurons (Saurat et al., 2013). By controlling subtypes within the progenitor pool, miRNAs help fine-tune their potencies so that multiple neuron types are produced sequentially and in the correct proportions.

During cortical neurogenesis, progenitors transition from a proliferative state—required for expanding and maintaining the progenitor pool—to a neurogenic state, where they must produce the correct numbers of neurons and lay the foundation for neuronal diversity. The principles of progenitor regulation by miRNAs may also be illustrated in systems outside the cortex, as described briefly in the following section.

### Neurogenesis in the Retina and Olfactory Bulb

Lineage relationships and neuronal diversity are relatively well-characterized in the retina and olfactory bulb. These systems provide additional examples of how miRNAs regulate progenitors to ensure that precise numbers and types of neurons are produced. miRNA ablation in retinal progenitors starting at E10.5 caused overproduction of early-born cell types (ganglion and horizontal cells) and failure to produce late-born cell types. These progenitors did not express late progenitor markers, reflecting a defect in progenitor developmental trajectory that ultimately disrupted neurogenesis (Georgi and Reh, 2010). This effect seems to be largely mediated by let-7, miR-125, and miR-9 repression of Prtg and Lin28b (La Torre et al., 2013). In the olfactory system, olfactory progenitor cells (OPCs) rely on a network of several basic helix-loop-helix (bHLH) transcription factors to generate olfactory sensory neurons representing a huge repertoire of olfactory receptors. miRNA ablation in OPCs downregulated key players such as Mash1, inhibited neurogenesis, and induced apoptosis of progenitors, all of which decreased the pool of sensory neurons. These defects were recapitulated by blocking the miR-200 family of five miRNAs (Choi et al., 2008). These examples show that miRNAs also play key roles regulating neurogenesis in the central nervous system beyond the cortex, sometimes utilizing common mechanisms observed in cortical neurogenesis.

After neurogenesis creates populations of immature neurons, more mechanisms involving miRNAs activate to reinforce neuronal fate and set up the molecular machinery underlying neuronal function.

### Determination of Neuronal Fate

As neurogenesis is underway, newly born neurons undergo further changes to embrace their neuronal fate, becoming post-mitotic and activating neuronal global gene expression programs. Neuronal fate determination relies heavily on the expression of miR-9 and miR-124, two of the most abundant and highly enriched miRNAs in neurons (Figure 1, top-left). Experiments in differentiating stem cells *in vitro* showed that manipulating these miRNAs changes the proportions of cells differentiating into neurons versus glia (Krichevsky et al., 2006). Ectopic expression of these miRNAs supports conversion of cultured fibroblasts into neurons, including development of neuron-like morphology, marker expression, and electrophysiological responses (Yoo et al., 2011). Neuronal fate determination involves global gene expression programs, so miR-9 and miR-124 must exert huge control over the transcriptional and regulatory landscape of cells. Both miRNAs repress specific subunits of the BAF chromatin-remodeling complex to induce epigenetic changes as part of a previously characterized, evolutionarily conserved neuronal development program (Yoo et al., 2011). They have also been suggested to cooperatively repress the REST transcription repressor complex to promote neuronal transcription programs and suppress the transition to gliogenesis (Krichevsky et al., 2006; Lee et al., 2018). Neurons use alternative splicing extensively to generate essential neuron-specific proteins and to target proteins to unique cellular compartments. PTBP1, a protein known to hinder neuron-specific alternative splicing, is repressed by miR-124 during early development to switch from general to neuron-specific alternative splicing programs, thus promoting neuronal differentiation (Makeyev et al., 2007). Adult neurogenesis in the subventricular zone (SVZ) produces olfactory bulb interneurons; differentiation of these cells from progenitors into neurons is also dependent on miR-124, which may target the stem cell maintenance gene Sox9 (Cheng et al., 2009).

Newly born neurons engage dramatic changes in gene expression and gene regulation to transform themselves from the progeny of stem-like progenitor cells into terminally differentiated neurons. miR-9 and miR-124 are key players in this process, working cooperatively to regulate influential transcriptional and post-transcriptional modifiers. Specification of neuronal fate then sets the stage for further differentiation into finer neuronal subtypes.

### Specification of Neuronal Subtypes

MicroRNAs reinforce additional fate decisions that allow neurons to differentiate into subtypes with distinct molecular characteristics. Among cortical PNs, corticospinal motor neurons (CSMNs) of layer V and callosal projection neurons (CPNs) of layers II/III and V form the corticospinal tract and corpus callosum, two main white matter tracts that are unique to placental mammals and that contribute to the functional and cell type complexity of these brains. miR-409-3p suppresses CPN fate in favor of CSMN development by downregulating LMO4, a transcription factor known to promote CPN areal identity. Layer V CSMN and CPN are born from the same progenitors and initially all express LMO4, so miR-409-3p is thought to mediate divergence of these two cell types (Diaz et al., 2020). Meanwhile, cortical inhibitory interneurons are born in several progenitor zones distant from the cortex and specify into different subtypes depending on which progenitor population they derived from. The medial ganglionic eminence (MGE) produces the cortical interneuron subtypes marked by expression of somatostatin (SST) or parvalbumin (PV). After miRNA ablation in all MGE cells, these interneurons failed to express SST or PV, indicating that miRNAs are necessary for interneuron subtype fate determination (Tuncdemir et al., 2015). While many of the miRNA-regulated molecular pathways regulating interneuron specification must still be characterized, it is clear that miRNAs are crucial for proper subtype specification.

There is also evidence that miRNAs help specify neuronal subtypes outside of the cortex. Olfactory sensory neurons produced by progenitors lacking DICER failed to express mature marker genes and olfactory receptors, indicating a differentiation defect in addition to decreased neurogenesis (Choi et al., 2008). The fate determination of spinal motor neurons (MNs) is also regulated by miRNAs. Generic spinal MN identity is established by cooperative binding of the LIM complex comprising ISL1 and LHX3 to MN-specific enhancers, thereby inducing the expression of a battery of MN genes that induce functional hallmarks of MNs, while suppressing key interneuron genes. The LIM complex highly and directly upregulates miR-218 at the onset of MN differentiation. miR-218 is specifically expressed in MNs throughout spinal cord development and is necessary for establishing MN fate while suppressing interneuron fate both *in vitro* and *in vivo* (Thiebes et al., 2015; Figure 1, bottom-right). miR-218 is also critical for mature MN maintenance and function, which we will discuss below. For a more detailed discussion on the role of miRNAs in the neurogenesis of spinal MNs we refer the readers to this review (Chen and Chen, 2019).

Together, these studies show that miRNAs coordinate progenitor proliferation and identity, neurogenesis, and adoption of neuronal fate. In doing so, they play critical roles in laying the groundwork for functional differentiation into neuronal subtypes and development of brain regions. We will now explore how miRNAs continue to guide cell type specification in postmitotic neurons by regulating migration and lamination, development of morphology and connectivity, and molecular identity.

## Migration and Lamination

Neuronal progenitors reside in bounded niches of the developing brain; to build other brain regions, they come in contact with neurons from other lineages, and form functional circuits, neurons must move out of progenitor zones after neurogenesis. Migration of newly born neurons must be precisely coordinated with other developmental processes, since migratory routes vary depending on cell identity and have been shown to influence morphological and functional development (Lim et al., 2018). For populations such as neural crest cells, it is known that migration along stereotyped routes helps resolve subtype identity, perhaps by bringing cells in contact with sequences of environmental signals (Soldatov et al., 2019). Lamination, the process of arranging cells in layers such as the six layers of cortex, relies on migration routes that specify final laminar positions. Cells in different layers may come from different lineages or birthdates, express distinct markers, form different connections and projections, and take on different microcircuit roles, meaning that lamination is closely tied up with cell identity. Defects in migration and lamination, as in the case of Type I lissencephaly, can induce additional phenotypes in morphology and firing properties (Ekins et al., 2020). Thus, migration, though a transient part of a neuron’s development, is a lasting influence on neuronal subtype diversity.

MicroRNA-mediated control of neuronal development includes migration and decisions made along the migratory path. In the cortex, DICER ablation in MGE-derived GABAergic interneurons induces a migration defect in addition to defects in mature marker expression. Interneurons must migrate tangentially from progenitor zones to reach and disperse throughout the cortex, after which they switch to radial migration and enter the cortical layers. Without miRNAs, many of these cells fail to enter the superficial migratory streams, causing a reduction in the number of interneurons reaching the cortex. These interneurons also show a defect in radially migrating into the cortical plate, contributing to reduced interneurons number across the cortical layers (Tuncdemir et al., 2015). However, the roles of specific miRNAs in this phenotype is still under investigation.

Meanwhile, excitatory pyramidal neurons (PNs) migrate radially from the ventricular progenitor zones. The combinatorial expression levels of miR-128, miR-9, and let-7b in radial glia modulate the lamination of the PNs they produce, including the proportions of cells that migrate into each layer. While miR-128 and let-7b seem to encode deep- and upper-layer identity in a dose-dependent manner, miR-9 specifically promotes development of layer V identity (Shu et al., 2019). Since the position of these cells in specific cortical layers is key to their function in stereotyped cortical microcircuits, this manipulation affects development of cortical cell types. Expression of miR-129-3p and miR-129-5p also controls migration across different PN identities. Upregulating or suppressing expression of these miRNAs causes PNs to fail to reach or overshoot their target layers, respectively, without affecting the expression of layer-specific marker genes. This effect is mediated by miR-129 repression of Fmr1, a gene associated with neurodevelopmental disorders. Cells overexpressing miR-129 were less likely to adopt a bipolar morphology, a key step preceding radial migration (Wu et al., 2019). Thus, miRNAs are able to control neuronal migration in coordination with layer identity, a key aspect of cortical cell type.

In some cases, we know that miRNAs directly regulate the molecular mechanisms of migration. Neurons follow fibers extended by radial glia across the cortical plate to climb into the cortical layers; they interact with these fibers through N-cadherin on the cell membrane. The miR-379/410 cluster regulates the expression of N-cadherin in both cortical progenitors and neurons. Simultaneously manipulating the expression of three miRNAs in this cluster—miR-396-3p, miR-496, and miR-543—reversibly controls the rate of migration out of the ventricular zone. Interestingly, overexpression of these miRNAs individually did not affect N-cadherin expression or induce a migration phenotype, but overexpressing pairs of miRNAs can bias neurons to migrate into the deep or upper layers (Rago et al., 2014). Elucidating the cooperative relationships between these miRNAs may further illustrate the complexity of miRNA network function in developing neurons. Meanwhile, altered miR-19 expression is associated with neurodevelopmental defects (Celli et al., 2003; Hemmat et al., 2014), suggesting that this miRNA plays an important role in neuronal development. While the mechanisms underlying the function of miR-19 in early development remain unclear, one study has found that miR-19 promotes migration of adult-born neurons born in the dentate gyrus, thus affecting their final position within the granule cell layer. miR-19 represses Rapgef2, a Rap1 and Rap2 activator known to influence cell adhesion and migration (Han et al., 2016).

MicroRNA control of migration, whether through affecting large programs coordinating layer identity or by directly affecting the molecular migration machinery, is an important contributor to the developmental trajectory of a cell. Laminar positioning then contributes to how a neuron integrates into circuits and takes on mature functions. Next, we continue along this developmental trajectory by examining miRNA functions in morphological and functional development.

## Morphology and Functional Connectivity

Neuronal morphology was historically the main way of categorizing cells and remains a key indicator of cell type and function. For example, parvalbumin (PV)-expressing interneurons share fast-spiking electrophysiological characteristics and of course expression of PV, but can be categorized further into basket cells, chandelier cells, and translaminar cells by their morphologies, which reflect diverging connectivity and circuit functions. By regulating the growth and elaboration of neuronal compartments, miRNAs control the development of cell type-specific characteristics.

### Cell and Neurite Morphology

MicroRNA function can have drastic effects on neurite outgrowth, and hence affect the function of individual cell types. This is evidenced by experiments where DICER was knocked out in postmitotic cortical pyramidal neurons. Loss of miRNAs caused dramatic reductions in soma size and neurite growth, which compounded into decreased brain size (Hong et al., 2013). miRNA ablation via knockout of Dgcr8, another component of the canonical miRNA biogenesis pathway, also in postmitotic pyramidal neurons revealed additional phenotypes in inhibitory synapse development: reduced PV interneuron abundance, reduced inhibitory synapse formation, and reduced IPSC amplitude and frequency (Hsu et al., 2012). DICER knockout in D1R-expressing striatal neurons also reduced cell size, leading to smaller brain size and profound defects in movement and behavior (Cuellar et al., 2008). Postnatal DICER knockout in cerebellar Purkinje cells caused gradual dendritic degeneration and spine loss, with eventual cell death and tissue degeneration (Schaefer et al., 2007). These dramatic phenotypes—from the neuronal level all the way to gross brain structure—suggest that miRNAs are crucial for multiple aspects of morphological development in neurons.

In addition, specific mechanisms mediated by individual miRNAs have been shown to regulate specific morphological characteristics such as dendritic outgrowth and maturation. miR-101 regulates dendrite development and scales overall network excitability through multiple parallel mechanisms. NKCC1 is a chloride channel that is downregulated as part of the GABA switch, when neuronal responses to GABA switch from excitation to inhibition. In this study blocking miR-101 repression of NKCC1 impaired neuronal functional maturation and increased spontaneous activity, which in turn increased dendritic growth and excitatory synapses. Derepression of other miR-101 targets, namely Ank2 and Kif1a, caused further hyperexcitability by producing and stabilizing more excitatory synapses (Lippi et al., 2016). miR-9 has key roles in neuronal fate determination; later in development, its repression of the REST complex also acts to increase dendritic length and complexity (Giusti et al., 2014). miR-132 and miR-212, contained in the same locus, have been shown to promote dendrite growth *in vitro* and in adult-born hippocampal neurons (Vo et al., 2005; Magill et al., 2010). In conjunction to its role in migration, miR-19 also regulates the development of mature morphology in adult-born hippocampal neurons. miR-19 overexpression led to decreased dendritic length and dendritic branching and prevented spines from becoming mature mushroom spines. Since miR-19 expression is high only in progenitor cells and turns off as neurons differentiate, it may control neuronal specification programs such as morphological maturation (Han et al., 2016).

Morphological development, including aspects of cell size, neurite growth, and spine formation, requires miRNA function. Elaboration of axons and dendrites must be coordinated with processes such as axon pathfinding, formation of specific synaptic connections, and circuit wiring, which will be explored in the following section.

### Functional Connectivity and Circuit Integration

Developing synaptic connections and integrating into circuits is fundamental to the functional maturation of any neuron. The arborization and targeting of dendrites and axons is specific to each cell type and carefully regulated during brain development. DICER knockout in dorsal root ganglion (DRG) neurons reduced axon growth *in vivo* and thus significantly reduced innervation of peripheral tissues. Inhibition of miR-132 phenocopies miRNA ablation *in vitro*. This effect has been suggested to be mediated by miR-132 repression of Rasa1, a Ras GTPase-activating protein known to respond to guidance cues, locally in axons (Hancock et al., 2014). Axon extension and targeting is critical to the functional development of retinal ganglion cells (RGCs), which must, in zebrafish, extend axons to the correct regions of the optic tectum. miR-182 regulates this process by repressing translation of Cfl1, part of the signaling cascade that responds to axon guidance cue Slit2 (Bellon et al., 2017). miR-409-3p promotes specification of CSMN fate over other projection neuron fates, and this includes the specific axon targeting of CSMNs. Overexpression of miR-409-3p caused more cells to not only express CSMN markers, but also to extend axons to the internal capsule, functionally joining the corticospinal tract (Diaz et al., 2020).

Olfactory interneurons provide a case where a specific miRNA has been shown to control the trajectory of a cell’s functional development, in a way that specifies distinct trajectories for developmentally- and adult-born neurons. The olfactory bulb contains interneurons born during early development in the olfactory bulb, and interneurons born during adulthood from subventricular adult progenitors. Early-born interneurons mature and integrate into circuits rapidly, while adult-born interneurons first migrate to the olfactory bulb and then slowly integrate into existing olfactory circuitry over several weeks. They form two distinct cell types, with different lineages, physiological characteristics, morphology, and roles in odor discrimination. miR-125 is expressed in only in the adult-born population, and inhibition of miR-125 leads to increased dendritic arborization in the olfactory bulb and abnormally early functional integration. This was assayed by quantifying *Fos* expression in 1-week-old interneurons following exposure to various odor stimuli. Thus miR-125 seems to control the timing of functional development in adult-born interneurons (Åkerblom et al., 2014; Figure 1, top-right).

In the cortex, perturbations in specification of one cell type can disrupt circuit formation, leading to additional abnormalities in other cell types and network-level defects. Cortical VIP-expressing interneurons disinhibit PNs by axonal targeting of other cortical interneurons. They play a key role in regulating network activity of cortical circuits, so disruption of VIP interneurons can negatively affect proper cortical function and development. VIP interneurons with postmitotic DICER ablation initially followed a normal developmental trajectory but exhibited progressive cell death in adulthood. Mature VIP interneurons also showed altered electrophysiological characteristics and deficits in synaptic inputs, leading to increased PN excitability and overall cortical activity (Qiu et al., 2020). These findings suggest that miRNAs are indispensable for maintaining the normal function of VIP interneurons, which have far-reaching effects on cortical circuit development and overall network function.

We have now seen that miRNAs guide newly born neurons through a complex series of developmental processes, including migration, morphological elaboration, and circuit integration. Next, we will examine how miRNAs interact with the transcriptomic environment of cells on unique developmental trajectories to resolve them into distinct cell types.

## Neuronal Subtype Determination and Function

In addition to their role in neural progenitor identity, cell fate determination and various developmental processes, miRNAs contribute to the diversity of cell types found in the nervous system. Not only do miRNAs exhibit developmental stage-specific expression patterns, but they also have distinct cell-type specific expression patterns. Indeed, miRNA expression profiles vary greatly across neurons and glial cell types including astrocytes, oligodendrocytes and microglia (Jovičić et al., 2013). Furthermore, from an unbiased screen in mouse, He et al. (2012) discovered that hundreds of miRNAs are enriched in different neuronal subtypes, such as cerebellar Purkinje neurons, cortical glutamatergic neurons, GABAergic INs, and even in different subtypes of INs. These findings strongly suggest an important role for miRNAs in neural cell type specification and maintenance. The identity of different neuronal and glial cell types is determined by combinatorial expression of transcription factors. Post-transcriptional regulation of their expression by miRNAs acts a network-level control mechanism that serves as a critical tuner of precise and robust identities. Below, we describe the specific miRNAs and their transcription factor targets involved in the determination of different neuronal subtypes such as dopaminergic (DA) neurons, spinal MNs and various sensory neurons.

### Dopaminergic Neurons

During development, transcription factors are known to operate in feedforward and feedback loops with miRNAs to reinforce lineage commitment. These are often negative feedback loops involving a cell type-specific miRNA, where the miRNA represses the transcription factor that induced its expression to prevent reverting to the previous developmental stage (Ebert and Sharp, 2012). For instance, miR-133b is specifically expressed in and regulates the maturation and function of midbrain DA neurons within a negative feedback loop that includes the transcription factor PITX3 (Figure 1, bottom-right). In this feedback loop, PITX3 specifically induces transcription of miR-133b, and miR-133b positively regulates dopaminergic neuron numbers in mouse primary midbrain cultures by downregulating PITX3 (Kim et al., 2007). In another example, a negative feedback loop involving the transcription factor ZEB2 and miR-200c was shown to control the expression and function of several key genes of midbrain DA neuron development (Yang et al., 2018; Figure 1, bottom-right). Among these genes was *Nr4a2*, which encodes for a transcription factor required for the generation of midbrain DA neurons. NR4A2 is also a known target of miR-132 (Yang et al., 2012; Figure 1, bottom-right). These examples highlight the specific miRNA/transcription factor loops involved in midbrain DA neuron differentiation.

The differentiation of olfactory bulb DA neurons is also under the regulation of a miRNA/transcription factor interaction. The transcription factor PAX6 is an important determinant of DA neurons in the olfactory bulb. In the postnatal and adult mouse forebrain, several olfactory bulb neuron subtypes are generated from a mosaic of neural stem cells that are spatially organized along the lateral ventricle. Olfactory bulb DA neurons are mainly generated from progenitors localized to the dorsal region of the ventricle. The mRNA of PAX6 is transcribed widely along the ventricular walls, but PAX6 protein expression is restricted to the dorsal region. This dorsal restriction was found to be a result of inhibition of PAX6 protein expression by miR-7a. Furthermore, *in vivo* inhibition of miR-7a in PAX6-negative regions of the lateral wall induces PAX6 protein expression and increased DA neuron number in the olfactory bulb (de Chevigny et al., 2012). Regulation of PAX6 by miRNAs has also been implicated in activity-dependent neurotransmitter switching between DA and GABA in developing *Xenopus* olfactory bulb interneurons in response to kinship odorants (Dulcis et al., 2017). This study found that miR-375 and miR-200b facilitate the switch between dopaminergic and GABAergic interneurons by targeting PAX6 and BCL11B, respectively. These findings highlight how regulation by miRNAs can alter neuronal identity.

### Motor Neurons

In addition to the differentiation of DA neurons in the midbrain and olfactory bulb, miRNAs have also been proven indispensable for the proper function of postnatal spinal MNs. In particular, a miRNA that is necessary to establish MN fate, miR-218, is also necessary for mature MNs (Figure 1, bottom-right). Mutant mice lacking miR-218 exhibit neuromuscular junction defects, MN hyperexcitability, and progressive MN cell loss (Amin et al., 2015). The relevance of miR-218 in proper function of MNs was further established with the discovery that miR-218 is downregulated in human amyotrophic lateral sclerosis (ALS). In this study the authors identified the potassium channel KV10.1 as a novel miR-218 target that regulates neuronal activity. From sequencing thousands of ALS genomes, they also identified six rare miR-218-2 gene variants that failed to regulate neuronal activity due to reduced processing by DICER, further highlighting the importance of miR-218 in MNs (Reichenstein et al., 2019).

### Sensory Neurons

Sensory neurons provide another set of examples, where cell type-specific miRNAs and their transcription factor targets interact to regulate neuronal subtype determination. The miR-183/96/182 cluster is highly expressed in sensory neurons and plays a role in regulating the molecular and functional identities of multiple different subtypes. Myelinated (A-fiber type) low-threshold mechanoreceptors (LTMRs) terminate peripherally in the skin and participate in touch sensation. There are three types of myelinated LTMRs: Aδ LTMRs, Aβ rapidly adapting LTMRs and Aβ slowly adapting (Aβ SA) LTMRs. Conditional loss of the miR-183/96/182 cluster in mice leads to a failure to extinguish expression of the transcription factor SHOX2 during development and an increase in the proportion of Aδ LTMRs at the expense of Aβ SA-LTMRs. Conversely, overexpression of the miR-183/96/182 cluster that represses SHOX2 expression, or loss of SHOX2, both increase the Aβ SA-LTMR population at the expense of Aδ LTMRs (Peng et al., 2018; Figure 1, bottom-right). Furthermore, the miR-183/96/182 cluster was shown to regulate the function of Aδ LTMRs by scaling acute noxious mechanical sensitivity by regulating CACNA2D1 and 2 and modulating neuronal excitability (Peng et al., 2017). The miR-183/96/182 cluster is also particularly important in photoreceptors, where it is highly expressed with peak levels in the adult retina. Cone-specific loss of miRNAs led to reduced expression of cone-specific genes and gradual degeneration of the outer segments, resulting in photoreceptors with reduced light responses (Figure 1, bottom-right). Re-expression of miR-183 and miR-182 prevented these phenotypes. The miR-183/96/182 cluster was also necessary and sufficient for the formation of inner segments, connecting cilia and short outer segments, as well as light responses in stem-cell-derived retinal cultures (Busskamp et al., 2014). The studies discussed above demonstrate the critical role of the miR-183/96/182 cluster in not only sensory neuron subtype determination but for their proper function. In addition, miRNAs were also found necessary for the retinal pigmented epithelium (RPE), which plays key supportive roles in photoreceptor development and function. Without miRNAs, RPE cells developed abnormal cellular morphology, underwent de-pigmentation, and showed defects in enzyme production, all of which contributed to non-cell-autonomous defects in photoreceptor differentiation (Ohana et al., 2015). In summary, in this section we have described how specific miRNAs can determine and maintain the identity of a wide variety of neuronal subtypes, through mechanisms that target a lineage’s unique transcriptome. Next, we discuss how miRNAs regulate various aspects of glial cell identity.

## Glial Subtype Determination, Morphology and Function

In recent years, numerous studies have demonstrated the importance of miRNAs in the development of various glial cell lineages such as Schwann cells (SCs) in the peripheral nervous system (PNS) and astrocytes, oligodendrocytes and microglia in the CNS. Below we summarize the critical miRNAs and their targets that regulate the differentiation, morphology, and function of these four glial subtypes.

### Microglia

Microglia are the resident macrophages of the CNS that represent the first line of immune defense in the brain and spinal cord. They originate from early yolk sac myeloid progenitors and infiltrate the brain during early development. In their steady state, microglia mainly function to maintain brain homeostasis and upon injury or infection microglia transform to an activated state. Emerging evidence continue to show that microglia are in fact a multifunctional housekeeping cell type that contributes to many aspects of brain development such as neural circuit wiring, neuronal survival, synaptogenesis, synaptic transmission and myelination (Thion and Garel, 2020; Cserép et al., 2021). Microglia-specific DICER ablation during embryonic development results in spontaneous microglial activation and accumulation of DNA damage (Varol et al., 2017). Consistent with this finding, there are numerous miRNAs known to inhibit microglia activation, which are described in detail here (Guo et al., 2019). Microglia are usually activated into two polarized states, termed the classical “M1” phenotype that releases destructive pro-inflammatory mediators and the alternative “M2” phenotype, which produces protective factors. Two neuronal enriched miRNAs, miR-128 and miR-124 is known to regulate microglia polarization. In a spinal cord injury (SCI) mouse model, miR-128 was downregulated in microglial cells and overexpression of miR-128 promoted viability of microglia and increased the expression levels of M2 phenotypic markers (Yang et al., 2017). miR-124 was also found to promote M2-like polarization by increasing the expression levels of M2 markers and decreasing M1 markers expression after injury (Hamzei Taj et al., 2016). It was further demonstrated that in a steady state, miR-124 can directly target C/EBP-α, a master transcription factor involved in differentiation of myeloid cells and its downstream target PU.1 and promote microglia quiescence (Ponomarev et al., 2011). Thus, miRNAs are critical for mediating microglial activation and polarization.

### Oligodendrocytes

Oligodendrocytes are a unique glial cell type in the CNS that synthesizes multilamellar myelin membranes which ensheath axons. Myelination electrically insulates axons to promote rapid, energy-efficient action potential propagation and is thus crucial for the development and function of the CNS. Two independent studies demonstrated that DICER ablation in oligodendrocyte precursor cells (OPCs) disrupts proper CNS myelination and delays oligodendrocyte differentiation (Dugas et al., 2010; Zhao et al., 2010). These studies identified miR-219 and miR-338 as oligodendrocyte-specific miRNAs and showed that overexpression of these miRNAs is sufficient to promote oligodendrocyte differentiation. miR-219 and miR-338 function in part by directly repressing negative regulators of oligodendrocyte differentiation SOX6, HES5, and ZFP238 (Figure 1, bottom-left). miR-7a is also highly enriched in OPCs, overexpression of which in NPCs or embryonic mouse cortex promoted oligodendrocyte generation. Blocking miR-7a function instead resulted reduction in oligodendrocyte number and an expansion of neuronal populations. miR-7a may exert these functions by directly targeting proneuronal transcription factors including PAX6 and NEUROD4 (Zhao et al., 2012; Figure 1, bottom-left). Finally, miR-23 has been shown to promote oligodendrocyte differentiation by downregulating Lamin 1B, a crucial determinant of oligodendrocyte maturation and myelin formation, whose overexpression was shown to suppress oligodendrocyte-specific genes (Lin and Fu, 2009; Figure 1, bottom-left). These studies together demonstrate the crucial role of miRNAs in promoting oligodendrocyte differentiation.

### Schwann Cells

Schwann cells are the principal glial cells in the PNS, where they play essential roles in the development, maintenance, function and regeneration of peripheral nerves. SCs can be categorized into two major classes - myelinating and nonmyelinating SCs. Myelinating SCs provide the myelin ensheathment of all large-diameter peripheral axons, while nonmyelinating SCs typically associate with smaller axons. Several studies have shown that DICER ablation in SCs fully arrests their differentiation and ability to myelinate. Gene expression analyses from these studies revealed drastic downregulation of promyelinating transcription factors KROX20 (Pereira et al., 2010) and EGR2 (Yun et al., 2010) and upregulation of myelination inhibitors NOTCH1 and HES1 (Pereira et al., 2010). Yun et al. (2010) also showed that DICER-ablated SCs maintained expression of genes characterizing the undifferentiated SC state, such as *Sox2, Jun, and Ccnd1*, providing some basis for the arrested differentiation of SCs. Further experiments will be required to determine the specific miRNAs and their targets that regulate SC differentiation and function (Bremer et al., 2010). Following axonal loss, SCs have the striking ability to dedifferentiate to an immature-like state, proliferate transiently, and help support axonal regeneration. As peripheral axons regenerate, SCs re-differentiate to form new myelin sheaths, helping to restore peripheral nerve function (Jessen and Mirsky, 2016). A miRNA profiling study in SCs following nerve injury found that most SC miRNAs were downregulated in response to injury, facilitating the de-differentiation process, which involves large-scale changes in gene expression. Shortly after axonal regrowth these expression levels of these SC miRNAs were restored. Most of these injury-regulated miRNAs were computationally predicted to target positive regulators of SC dedifferentiation and proliferation that must be repressed for proper SC re-differentiation to occur. In particular, the authors found that miR-34a targets NOTCH1 and CCND1, and miR-140 targets EGR2 to modulate dedifferentiation/proliferation and remyelination, respectively (Viader et al., 2011; Figure 1, bottom-left). Altogether these studies indicate that miRNAs are critical for facilitating SC differentiation and maintaining the differentiated state of mature SCs.

### Astrocytes

Astrocytes are the most abundant cell type in the CNS and are crucial regulators of neuronal development, function and connectivity. Thus, it is not surprising that astrocyte-specific DICER ablation results in drastic phenotypes across multiple regions in the brain. These phenotypes range from deficits in dendritic spine development in cortical and hippocampal neurons (Sun et al., 2019) to major behavioral phenotypes such as ataxia, seizures and ultimately premature death (Tao et al., 2011). The cerebellum was found to be particularly susceptible to insults caused by astrocytic DICER ablation, largely due to dysfunction of Bergmann glia (BG), specialized astrocytes that serve as scaffolds during early postnatal cerebellar development. DICER-ablated BG exhibited thickened, swollen processes preceding cerebellar degeneration and ataxia, suggesting that miRNAs have a direct role in the morphology and function of BG (Tao et al., 2011). A different study showed that, in addition to morphological defects, embryonic DICER ablation in astrocytes results in decreased expression of BG markers and an intrinsic blockage of Notch signaling. Notch signaling is crucial for the maintenance of BG during postnatal cerebellar development. Furthermore, the authors found that miR-9 is required for proper Notch1 signaling in early postnatal BG (Kuang et al., 2012). Recent studies have implicated more miRNAs in astrocyte development and function. miR-31, a miRNA enriched in differentiated astrocytes, is required for terminal astrocyte differentiation as the loss of miR−31 impairs differentiation and prevents astrocyte maturation *in vitro* (Figure 1, bottom-left). This function is mediated in part by miR-31 targeting of LIN28, a stem cell factor implicated in neural progenitor cells (Meares et al., 2018). Another *in vitro* study showed that miR-125b positively regulates astrogliogenesis and promotes astrocyte proliferation (Pogue et al., 2010; Figure 1, bottom-left). Finally, a FMRP-dependent miRNA-mediated mechanism has been shown to alter developmental astroglial mGluR5 signaling, which is important for mediating developmental astroglia to neuron communication. Selective loss of FMRP in mouse and human astroglia *in vivo* was shown to upregulate a brain-enriched miRNA, miR-128-3p, which in turn suppressed developmental expression of astroglial mGluR5. mGluR5 expression was not altered in FMRP-deficient neurons, highlighting an astroglia-specific mechanism (Men et al., 2020). These studies demonstrate the crucial role of miRNAs in of astrocyte differentiation, morphology and function. Overall, we have outlined how miRNAs are critically involved in various developmental processes of distinct glial subtypes found in the nervous system.

## Cell Maintenance and Survival

Seeing as miRNAs are necessary for developing the characteristics of cell type—including laminar position, morphology, connectivity, and molecular markers—it is not surprising that they also play key roles in maintaining these cell types. Differentiated neurons must tightly regulate gene expression to remain functional throughout life and avoid cell death. Continual miRNA regulation buffers potentially harmful changes in gene expression and stabilizes a cell’s identity over its lifetime. Decreased miRNA function, by allowing harmful changes in identity and gene expression networks, may contribute to neurodegeneration. Recent studies have also suggested that many miRNAs repress pro-apoptotic genes, such that miRNA misexpression or gain-of-function mutations may provide cancerous cells with a mechanism of escaping apoptosis, making miRNA dysfunction a potential “second hit” during oncogenesis (Bejarano et al., 2021). The following examples will highlight degenerative phenotypes caused by miRNA dysfunction in the brain, in glial cells, and in other CNS neurons, such as those of the spinal cord.

Reduced miRNA function caused by hemizygous DICER knockout in adult DA neurons reduced levels of DA and other molecules in DA metabolic pathways, while complete DICER knockout led to severe DA neuron death, especially in the substantia nigra (Chmielarz et al., 2017). Dysfunction and progressive loss of DA neurons underlies Parkinson’s disease (PD), leading some to hypothesize that age-related decline in miRNA function may contribute to PD pathology. miRNA biogenesis has been observed to decline with age, and pharmacological stimulation of miRNA biogenesis improved DA neuron survival after cellular stress. Downregulation of midbrain-enriched miRNAs, such as miR-133b, may contribute to the selective vulnerability of DA neurons (Kim et al., 2007; Figure 1, top-right). miRNA ablation in cerebellar Purkinje cells eventually leads to cell death following morphological degeneration (Schaefer et al., 2007), and MGE-derived GABAergic interneurons also show increased apoptosis after DICER knockout (Tuncdemir et al., 2015). A specific subtype of interneurons, VIP interneurons were also found to exhibit progressive cell death with postmitotic DICER knockout (Qiu et al., 2020).

During development, oligodendrocytes are greatly overproduced and only by competing for limiting amounts of target-derived growth factors is their number adjusted according to the number and length of the axons requiring myelination. The miR-17/92 cluster was shown to regulate this culling process of oligodendrocytes by promoting their proliferation through activation of the Akt signaling pathway (Budde et al., 2010). Postnatal ablation of DICER in microglia found that absence of miRNAs does not induce spontaneous activation or morphological abnormalities but results in reduced microglia number, highlighting a role for miRNAs in microglia survival during adulthood (Varol et al., 2017).

Without miR-218-1 and miR-218-2, spinal MNs are born in normal numbers but fail to innervate muscles and die off in large numbers before birth (Amin et al., 2015; Figure 1, top-right). Proprioceptive DRG neurons express PV late in development, meaning that conditional DICER knockout driven PV expression ablates miRNAs in only more mature cells. These mutant mice initially develop normally, with normal proprioceptive DRG neuron number and function, but show progressive proprioceptive DRG neuron loss and locomotor defects by P30. Cell death is preceded by disruption of cell identity, as evidenced by downregulation of proprioceptor-enriched genes, upregulation of markers for other cell types, and decreased responsiveness to vibration (O’Toole et al., 2017). DICER knockout in mature, postmitotic rod photoreceptors lead to outer segment disorganization in adult mice, followed by robust retinal degeneration and eventual loss of visual function. Interestingly, these mice did not exhibit significant defects in either phototransduction or the visual cycle prior to retinal degeneration, suggesting that miRNAs in rods primarily function to support their survival (Sundermeier et al., 2014).

In addition to developmental establishment of cellular diversity, miRNA function throughout life is necessary to maintenance of cell identity and health. Dysfunction of miRNA networks can induce degenerative phenotypes and may contribute to the mechanisms or the cell type-specificity of some neurodegenerative diseases.

## Discussion

Numerous reports over the last two decades have shown that miRNA-mediated posttranscriptional regulation is indispensable at basically all stages of brain assembly. Although the majority of these studies are focused on developmental transitions, here we argue that the same mechanisms also demonstrate that miRNAs are key determinant of cell identity. By reinforcing the transcriptional programs underlying lineage decisions, miRNAs instruct the emergence and maintenance of many cell types of the brain. Further, miRNAs are necessary building blocks in the molecular pathways controlling lamination, differentiation and proper connectivity, processes through which neurons take on the characteristics essential to their cell type. Despite this wealth of data, we believe that the field is still in its infancy. In fact, miRNA roles have only been tested in a small percentage of brain cell types. Even for the ones tested, detailed miRNA mechanisms are for the most part still missing. Finally, owing to advances in sequencing technologies, the field is rapidly progressing from studying a single miRNA and a few of its targets to identifying miRNA-target networks associated with distinct cell types and developmental stages (Nowakowski et al., 2018). This effort is complemented by new technologies that allow precise mapping of miRNA-target interactions (mTI) in specific cell types (Tan et al., 2013; Li et al., 2020). Integrating miRNA and target expression profile with mTI mapping will deliver, for the first time, the landscape of miRNA repression in specific cell types. The necessary next step will be to develop scalable tools that effectively manipulate mTI so that their functional roles in instructing cell identity can be tested. The combination of these technologies will reveal novel mechanisms driving cellular diversity in the brain and spawn a new era of miRNA research.

## Author Contributions

NZ and JD wrote the review with help from FZ and GL. All authors contributed to the article and approved the submitted version.

## Conflict of Interest

The authors declare that the research was conducted in the absence of any commercial or financial relationships that could be construed as a potential conflict of interest.
